# Retraction Note: Quercetin-induced miR-200b-3p regulates the mode of self-renewing divisions in pancreatic cancer

**DOI:** 10.1186/s12943-023-01834-7

**Published:** 2023-08-04

**Authors:** Clifford C. Nwaeburu, Alia Abukiwan, Zhefu Zhao, Ingrid Herr

**Affiliations:** Department of General, Molecular OncoSurgery, Section Surgical Research, Visceral and Transplantation Surgery, University of Heidelberg, Im Neuenheimer Feld 365, 69120 Heidelberg, Germany

**Retraction note: Mol Cancer 16, 23 (2017)**

10.1186/s12943-017-0589-8

The authors have retracted this article because there are overlaps in three figures. Specifically, in Figure 6A, in column 3, two panels are identical but are described as deriving from different cell lines AsPC1 and AsanPaCa; additionally, there is overlap between two panels for AsanPaCa in columns 1 and 2 despite each panel corresponding to different treatments; finally, there is overlap between two panels for PANC1 in columns 3 and 4 despite each panel corresponding to different treatments. In Figure 6B, there is overlap between two panels for AsanPaCa in columns 1 and 2 despite each panel corresponding to different treatments. In Figure 6C, there is overlap between two panels for AsPC1 in columns 3 and 4 despite each panel corresponding to different treatments.

All authors agree to this retraction.

